# Identification of Genetic Variants in Status Epilepticus Associated With Fever

**DOI:** 10.1002/brb3.70279

**Published:** 2025-02-06

**Authors:** Hiroaki Hanafusa, Hiroshi Yamaguchi, Naoya Morisada, Ming Juan Ye, Shizuka Oikawa, Shoichi Tokumoto, Masahiro Nishiyama, Kandai Nozu, Hiroaki Nagase

**Affiliations:** ^1^ Department of Pediatrics Kobe University Graduate School of Medicine Hyogo Japan; ^2^ Department of Genetics Hyogo Prefectural Kobe Children's Hospital Hyogo Japan; ^3^ Department of Neurology Hyogo Prefectural Kobe Children's Hospital Hyogo Japan

**Keywords:** children, epilepsy, gene panel, microarray, seizure, target gene sequencing, whole exome sequencing

## Abstract

**Purpose:**

Status epilepticus associated with fever (SEF) is often encountered in pediatric emergency departments, and some patients develop neurological emergencies, such as acute encephalopathy (AE). Although numerous genetic variants of developmental and epileptic encephalopathy (DEE) have been reported, the frequency of these disease‐associated variants of SEF is unknown. The first aim of this study was to investigate the associated genetic variants of SEF. The second aim was to compare the variations in genes between SEF and DEE.

**Method:**

This retrospective, clinical observational study included patients with SEF or DEE who visited Kobe University Hospital or Kobe University affiliated hospitals and provided consent for a genetic diagnosis of SEF or DEE between January 1, 2021, and December 31, 2022.

**Finding:**

Fifteen patients with SEF and 27 patients with DEE consented to a genetic diagnosis and were included in the study. The detection rate of genetic variants was lower in patients with SEF (26.7%) than in those with DEE (63.0%), although there is no statistically significant difference (*p* = 0.05, Fisher's exact test). Analysis of patients with DEE revealed a wide variety of causative genes for DEE (16 different genes), whereas in SEF cases, only *SCN1A* variants were detected.

**Conclusion:**

Our study is the first to clarify the detection rates of different genetic variants in SEF. Patients with SEF may have less genetic involvement in the onset of epileptic seizures, compared to those with DEE.

## Graphical abstract

The detection rate of genetic variants was lower in patients with status epilepticus associated with fever (SEF) (4/15; 26.7%) than in those with DEE (17/27 63.0%). DEE revealed a wide variety of causative genes (16 different genes), whereas in SEF cases, only *SCN1A* variants were detected. SEF and DEE may have different onset mechanisms.

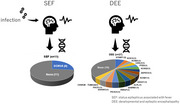

## 1 Introduction

**FIGURE 1:**
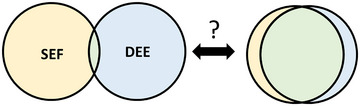
With regard to the patients’ monogenic backgrounds of SEF and DEE, a Venn diagram is employed to illustrate the overlap of single gene variants. The left diagram represents scenarios where there is minimal sharing of background genetic variants between SEF and DEE. Conversely, the right diagram illustrates situations where the single gene variants in SEF and DEE are nearly identical.

Febrile seizure (FS) is the most common form of infection‐triggered seizures among children, affecting 2%–5% of those aged between 6 months and 6 years (Nishiyama et al. 2020; Subcommittee on Febrile Seizures, American Academy of Pediatrics 2011). Status epilepticus associated with fever (SEF) is often encountered in the pediatric emergency department, and most cases of SEF are caused by prolonged FS without neurological sequelae, where only the seizure is managed. However, some patients with SEF develop neurological emergencies, such as acute encephalopathy (AE; Hayakawa et al. 2016). In some cases, longer seizure duration can be a sign of AE, (Tang et al. 2024) and SEF can occur in the context of AE (Takanashi 2009). Therefore, SEF and AE are closely related. AE occurs in 400–700 children in Japan annually, and its incidence is reportedly high, especially in East Asia, suggesting a genetic background. However, infections (primarily viral, bacterial, or mycoplasmal) are the leading cause of AE in children (Mizuguchi et al. 2007, 2021). Most patients with SEF tend to have a favorable outcome without lasting sequelae. While most patients with SEF tend to have favorable outcomes without long‐term sequelae, the prognosis significantly differs when seizures occur in the context of AE, compared to those associated with FS. Many healthcare professionals may ponder whether such cases have any genetic predisposition or if there exists a shared underlying genetic background, involving a monogenic etiology common to developmental and epileptic encephalopathies (DEEs; Figure 1). In 2017, the International League Against Epilepsy introduced the concept of DEE (Scheffer et al. 2017). DEE is characterized by encephalopathy with intellectual disabilities associated with developmental delay or epilepsy, which is often resistant to antiseizure medicine. Genetic variants are often found in patients with DEE, owing to recent advances in genetic testing (Bartolini 2021).

To the best of our knowledge, no study has identified the different variants of background genes in patients with SEF. This may be attributed to the fact that many patients progress without sequelae, leading parents to decline genetic testing, or because FSs are relatively rare outside regions, such as East Asia, resulting in an insufficient number of cases to justify such testing.

First, in this study, we aimed to investigate disease‐associated gene variants in patients who have not yet been diagnosed with epilepsy and who developed SEF from fever‐induced seizures, as well as in those with DEE without a history of SEF. Second, we sought to compare the genetic variants between these two groups to elucidate the differences in the influence of each genetic variant on disease etiology.

## 2 Materials and Methods

### 2.1 Study Design and Patients

This retrospective clinical observational study was approved by the Ethics Committee of Kobe University (2022‐86). All the experiments were performed in accordance with the relevant guidelines and regulations of these institutions and the Code of Ethics of the World Medical Association (Declaration of Helsinki). Written informed consent was obtained from the parents of all patients. As of January 1, 2021, our department initiated genetic testing for patients with a medical history of SEF and DEE. The selection of genetic tests was determined by the attending physician, considering the patient's medical history, family history, and clinical findings. In this study, we retrospectively reviewed the medical charts of patients with a medical history of SEF and those with DEE who visited Kobe University Hospital and Kobe University‐affiliated hospitals (Hyogo Prefectural Kobe Children's Hospital and Takatsuki General Hospital) between January 1, 2021, and December 31, 2022, and requested a genetic diagnosis of SEF and DEE. We collected data on demographics, clinical presentation, treatments, imaging findings, and laboratory tests of these patients.

### 2.2 Definitions

FS was defined as seizures accompanied by fever (temperature ≥ 100.4°F or 38°C), without central nervous system infection, in infants and children aged 6–60 months, according to the American Academy of Pediatrics (Subcommittee on Febrile Seizures, American Academy of Pediatrics 2011). Seizure duration was defined as the time from seizure onset (according to information provided by caregivers) to the cessation of seizures (as confirmed by information provided by caregivers or emergency department physicians; Yamaguchi et al. 2018, 2020). SEF was defined as a seizure lasting for ≥ 30 min or a recurrent seizure lasting for ≥ 30 min without full recovery of consciousness triggered by fever. Although SE has previously been defined as a seizure or a series of seizures lasting ≥ 30 min (Berg et al. 2004), seizures lasting for 5 min have recently been classified as an operational definition of SE because such seizures often do not cease spontaneously (Brophy et al. 2012). In our study, a seizure duration of ≥ 30 min was adopted as SE because of the increased risk of mortality (Cheng 2016). “Febrile” was defined as a temperature of > 38°C (Yamaguchi et al. 2018). The diagnosis of AE was based on the Japanese Clinical Consensus and Guidelines for Acute Encephalopathy in Children (Committee of Guidelines for Acute Encephalopathy in Children 2016). In brief, AE was diagnosed when a patient had disturbed consciousness with an acute onset lasting at least 24 h (Committee of Guidelines for Acute Encephalopathy in Children 2016). Patients with obvious central nervous system infections, such as meningitis or encephalitis (defined by a cerebrospinal fluid cell count > 8 cells/µL), were excluded from the study (Committee of Guidelines for Acute Encephalopathy in Children 2016; Oikawa et al. 2024). In this study, SEF cases were defined as those presenting with status epilepticus induced by fever (suspected infection) and not yet diagnosed with DEE. In contrast, patients with DEE had never experienced status epilepticus during febrile episodes.

The term “DEE” includes developmental impairment related to both the underlying etiology, independent of epileptiform activity, and the epileptic encephalopathy (Riney et al. 2022). In this study, DEE was also defined as cases with no history of SEF. The diagnosis of epilepsy was based on the 2014 definition by the International League Against Epilepsy (Fisher et al. 2014), which includes the following criteria: (1) the occurrence of two or more unprovoked seizures more than 24 h apart; (2) the occurrence of one unprovoked seizure with a recurrence risk over the next 10 years comparable to the general risk after two unprovoked seizures; or (3) a diagnosis of an epilepsy syndrome.

Neurological performance at baseline and prognosis after onset were assessed using the Pediatric Cerebral Performance Category scale. On this scale, scores of 1, 2, 3, 4, 5, and 6 indicate normal function, mild impairment, moderate impairment, severe impairment, prolonged vegetative state, and death, respectively (Fiser 1992).

### 2.3 Commercial‐Based Genetic Testing

Patients 5, 7, and 30 were clinically suspected of having Dravet syndrome, and commercial gene panel testing was performed at the Kazusa DNA Research Institute (https://www.kazusa.or.jp/genetest/). This gene panel included *SCN1A, SCN1B, SCN2A*, and *GABRG2*.

### 2.4 Direct Sequencing and Subcloning

Patient 28 was clinically suspected of having *PIK3CA*‐related overgrowth syndrome, and direct sequencing was performed. Genomic DNA was extracted from the peripheral blood of the parents using the QuickGene‐Auto S DNA Blood Kit (Kurabo Industrial Ltd., Osaka, Osaka, Japan). Polymerase chain reaction (PCR) was performed using Tks Gflex DNA Polymerase (Takara Bio Inc., Kusatsu, Shiga, Japan), and direct sequencing was performed using the BigDye Terminator v1.1 Cycle Sequencing Kit and Applied Biosystems SeqStudio Genetic Analyzer (Thermo Fisher Scientific, Waltham, MA). The primers used for PCR and direct sequencing are listed in Table S1. A mosaic variant was suspected in *PIK3CA* based on a sequence electropherogram, and subcloning analysis was performed. PCR products obtained using the HotStarTaq Plus Master Mix Kit (Takara Bio Inc.) were ligated into the pT7Blue T‐Vector (Novagen, Madison, WI) using a DNA Ligation Kit Ver. 2.1 (Takara Bio Inc.), and subcloning was performed using *Escherichia coli* HST08 Premium Competent Cells (Takara Bio Inc.). Colonies were selected using ampicillin and X‐gal, followed by colony PCR performed using the EmeraldAmp PCR Master Mix (Takara Bio Inc.) and primers (F: 5’‐TAATACGACTCACTATAGGG‐3’, R: 5’‐GTTTTCCCAGTCACGACGTTG‐3’). PCR products were analyzed by electrophoresis on a 1.5% agarose gel and direct sequencing with a BigDye Terminator v1.1 Cycle Sequencing Kit and Applied Biosystems SeqStudio Genetic Analyzer (Thermo Fisher Scientific).

### 2.5 Original Panel Sequencing

Genomic DNA was extracted from the peripheral blood of the parents using the QuickGene‐Auto S DNA Blood Kit (Kurabo Industrial Ltd.). The library was prepared with SureSelect PostPool Custom Tier2 (0.5–2.9 Mb) (Agilent Technologies, Santa Clara, CA). The 331 genes included in the panel have been previously reported (Oikawa et al. 2024) and are listed in Table S2. Sequencing was performed using the MiSeq platform (Illumina, San Diego, CA), and analysis was performed using SureCall v4.2.1.10 software (Agilent Technologies). Direct sequencing was performed to confirm candidate variants, which were evaluated according to the American College of Medical Genetics and Genomics (ACMG) and the Association for Molecular Pathology (AMP) guidelines (Richards et al. 2015). Segregation analysis was performed by direct sequencing using gDNA derived from parents’ blood to confirm the presence of variants revealed in the probands. Therefore, the test for fatherhood was not performed.

### 2.6 Whole Exome Sequencing (WES)

Genomic DNA was extracted from the peripheral blood of the parents using the QuickGene‐Auto S DNA Blood Kit (Kurabo Industrial Ltd.). Whole‐exome sequencing was performed using NovaSeq 6000 (Illumina) and SureSelect Human All Exon V6 (Agilent Technologies). Reads were mapped to human genome assembly hg38 with the BWA‐MEM algorithm of Burrow–Wheeler Aligner v0.1.17 (Li and Durbin 2009). To obtain BAM files, PCR duplicates were marked with Picard MarkDuplicate v2.18.29 (http://broadinstitute.github.io/picard), and the base quality score was recalibrated using the Genome Analysis Toolkit (GATK) v4.3.0.0 (McKenna et al. 2010). Variant calling was then performed using the GATK Haplotype caller, and VCF files were obtained. The VCF files were annotated using ANNOVAR v2019Oct24 (Wang, Li, and Hakonarson 2010). Direct sequencing was performed to confirm the candidate variants found by WES, and the variants were evaluated according to the ACMG/AMP guidelines (Richards et al. 2015). Segregation analysis was performed by direct sequencing using gDNA derived from parents’ blood to confirm the presence of variants revealed in the probands. Therefore, the test for fatherhood was not performed. In patient 24, a variant was detected at the splice site of *DEPDC5*. Total RNA was extracted from the patient's blood using RiboPure RNA Purification Kit, blood (Thermo Fisher Scientific) and cDNA was obtained with SuperScript III Reverse Transcriptase (Thermo Fisher Scientific). Direct sequencing was performed on the obtained cDNA using Tks Gflex DNA Polymerase, BigDye Terminator v1.1 Cycle Sequencing Kit, and Applied Biosystems SeqStudio Genetic Analyzer to confirm the effect on splicing.

### 2.7 Microarray

Comparative genomic hybridization and single‐nucleotide polymorphism arrays were performed using GenetiSure Dx Postnatal Assay (Agilent Technologies). A SureScan Dx Scanner (Agilent Technologies) was used for scanning, and CytoDx (Agilent Technologies) software was used for analysis.

### 2.8 Statistics

The results are expressed as numbers. Fisher's exact test was used as appropriate to analyze the data. All analyses were performed using GraphPad Prism 5.0 (GraphPad Software, San Diego, CA). Statistical significance was set at *p* < 0.05.

## 3 Results

### 3.1 Clinical Background of the Patients With SEF and DEE

The clinical backgrounds of patients with SEF and DEE are shown in Tables 1 and 2, respectively. Fifteen patients were diagnosed with SEF (Table 1). The sex ratio was 9:6 (male:female). The median age at onset was 18 months (interquartile range [IQR], 13–37 months). Out of 15 patients diagnosed with SEF, five were finally diagnosed with AE (AESD, *n* = 3; hemorrhagic shock and encephalopathy syndrome, *n* = 2). The etiology of infection was identified in five cases. Generalized tonic‐clonic seizures (nine patients [60.0%]) and focal‐to‐bilateral tonic‐clonic seizures (three patients [30.0%]) were common types of seizures. The median duration of seizures was 120 (IQR, 62–168) min. Twenty‐seven patients were diagnosed with DEE (Table 2). The sex ratio was 14:13 (male:female). The age at onset was < 1 year in more than half of the patients with DEE (63.0%). Most patients had developmental delays or intellectual disabilities.

**TABLE 1:** Clinical background of the patients with SEF.

Patient no.	Sex	Age at onset	Diagnosis	Etiology	Seizure type	Seizure duration (min)	Prognosis (PCPC)	Birth history	Developmental history	Past medical history	Family history	Brain imaging findings	Age at genetic test	Treatment at onset
Patient 1	M	1y10m	SEF	Unknown	Tonic	158	1 (prior to admission) 2 (at discharge) 1 (1m) 1 (6m) 1(1y)	GA: 39w2d BW: 2886 g HT: 152 cm HC: 32.5 cm	No developmental delay in onset	None	Autism (brother)	CT (day 1): normal MRI (day10): normal	1y11m	DZPsp DZPiv fPHTiv PBiv TTM+BCT
Patient 2	F	1y11m	SEF	Parainfluenza3	GTCS	60	1 (prior to admission) 1 (at discharge) 1 (6m), 1 (1y)	GA: 41w0d BW: 2560 g	Generalized developmental delay, start to walk (1y9m)	None	FS (grandfather, grandmother)	CT (day1): subdural hematoma in the right parietal legion MRI (day8): right parietal, temporal and occipital hyperintensity (T1WI)	2y	MDLiv fPHTiv TTM+BCT
Patient 3	M	3m	AE (HSES)	Unknown	Tonic	149	1 (prior to admission) 4 (at discharge) 4 (6m) 4 (1y) 4 (2y)	GA: 40w0d BW: 2900 g	No developmental delay in onset	None	None	MRI (day14): cerebellar hemispheres and cerebral hemispheres show globally hypointensity (T1WI), hyperintensity (T2WI), hypointensity (FLAIR). Hyperintensity in the cortex and subcortical white matter, including the occipital and parietal lesions, and bilateral basal ganglia (DWI). Hyperintensity suggesting hemorrhagic necrosis in bilateral parietal and occipital gyri (T1WI)	5y	MDLiv, MDLcont PBiv SPT intubation
Patient 4	F	1y2m	SEF	Unknown	FBTCS	231	1 (prior to admission) 2 (at discharge) 2 (1m), 3 (1y)	GA: 38w5d BW: 3062 g cesarean section because of breech position	Mild verbal developmental delay (1y3m), ESID: DQ78 (2y2m)	Afebrile seizures after vaccination (4m)	None	MRI (day9): normal	1y3m	MDLiv fPHTiv PBiv Thiiv TTM+BCT
Patient 5	F	2y7m	SEF	Adenovirus (rapid antigen test)	GTCS	340	2 (prior to admission) 2 (at discharge) 2 (1m) 3 (6m) 3 (1y) 3 (2y)	GA: 37w1d BW: 2861 g	No developmental delay in onset	Afebrile seizure (FIAS)(3m), FS (4m,5m,6m)	Developmental delay (brother)	CT (day1): normal, MRI (day10): normal	2y7m	MDLom DZPsp DZPiv fPHTiv PBiv TTM+BCT
Patient 6	F	5y11m	SEF	Unknown	Tonic	165	1 (prior to admission) 1 (at discharge) 1 (1m) 1 (6m) 1 (1y)	GA: 40w4d BW: 3495 g cesarean section	No developmental delay in onset	Afebrile seizure (4m), recurrent FSs	FS (mother)	CT (day1): normal, MRI (day8): normal	6y	DZPiv, fPHTiv PBiv TTM+BCT
Patient 7	F	８m	SEF	Unknown	FBTCS	45	1 (prior to admission) 1 (at discharge) 1 (1m)	GA: 40w0d BW: 3620 g	No developmental delay in onset	Unilateral afebrile seizures (6m), FS (8m)	None	MRI (day13): mild degree of cerebral atrophy	8y	MDLdiv TTM+BCT
Patient 8	M	1y0m	SEF	Unknown	FBTCS	30	1 (prior to admission) 1 (at discharge) 1 (6m) 1 (1y)	GA: 38w0d BW: 2788 g	No developmental delay in onset	Microcephaly, Hypertrophic pyloric stenosis (2m), recurrent FS	Hyperthyroidism (mother)	None	1y5m	DZPsp
Patient 9	M	4y0m	AE (HSES)	Unknown	GTCS	120	2 (prior to admission) 5 (at discharge) 5 (6m) 5 (1y) 5 (2y)	GA: 37w0d BW: 2610 g	Language delay (2y4m), generalized delay (3y)	Recurrent FS	None	MRI (day2): hyperintensity throughout the cerebrum (DWI)	6y	None
Patient 10	F	3y8m	AE (AESD)	Unknown	GTCS	171	3 (prior to admission), 4 (at discharge)	GA: 37w3d BW: 2114 g	Without significant words, up to sitting position (3y)	Small jaw, early neonatal hypotonia, hypothyroidism	None	MRI (day6): BTA	6y	MDLiv Thiiv TTM+BCT, SPT
Patient 11	M	3y6m	AE (AESD)	Unknown	GTCS	30	2 (prior to admission), 2 (at diacharge), 2 (1m) 2 (3m) 2 (6m) 2 (1y) 2 (2y)	GA: 37w5d BW: 2569 g	No significant words (3y)	Developmental delay	None	MRI (day10): BTA	14y	DZPsp
Patient 12	M	1y5m	SEF	Unknown	GTCS	249	1 (prior to admission), 1 (at discharge), 1 (1m) 1 (3m) 1 (6m)	Unknown	No developmental delay in onset	None	None	CT (day1): normal, MRI (day8): normal	１y5m	DZPiv MDLiv fPHTiv mitochondria rescue SPT TTM+BCT
Patient 13	M	9m	SEF	HHV6 (cerebrospinal fluid)	GTCS	64	1 (prior to admission), 1 (at discharge), 1 (2m)	GA: 38w3d BW: 2698 g	Developmental delay (borderline)	Developmental delay	None	CT (day1): normal, MRI (day11): normal	9m	MDLiv DZPiv, MDLcont, TTM+BCT
Patient 14	M	1y2m	AE (AESD)	HHV6	GTCS	90	1 (prior to admission), 4 (at discharge), 4 (6m)	GA: 38w4d BW: 2805 g	No developmental delay in onset	None	None	CT (day1): global brain edema, MRI (day7): BTA, MRI (3m): Mild to moderate atrophy of the entire cerebrum	1y3m	DZPiv fPHT, MDLcont, SPT
Patient 15	M	1y6m	SEF	Adenovirus, rhinovirus/ enterovirus, coronaHKU1, parainfluenza3	GTCS	81	1 (prior to admission), 1 (at discharge), 1 (6m)	GA: 39w5d BW: 3356 g	No developmental delay in onset	None	FS (father&mother)	CT (day1): normal, MRI (day8): normal	1y8m	MDLiv fPHTiv Thiiv, TTM+BCT

Abbreviations: AE, acute encephalopathy; AESD, acute encephalopathy with biphasic seizures and late reduced diffusion; BTA, bright tree appearance; BW, body weight; cont, continuous; CT, computed tomography; diazepam; DQ, Developmental; DWI, diffusion weighted imaging; DZP; ESID, Enjoji Scale of Infant Analytical development; F, female; FBTCS, focal to bilateral tonic‐clonic; FIAS, focal impaired awareness seizures; FLAIR, fluid attenuated inversion recovery; fPHT, fosphenytoin; FS, febrile seizure; GA, gestational age; GTCS, generalized tonic‐clonic seizure; HC, head circumference; HHV, human herpesvirus; HKU, human coronavirus; HSES, hemorrhagic shock and encephalopathy syndrome; HT, height; iv, intravenous; M, male; m, month; MDL, midazolam; min, minute; MRI, magnetic resonance imaging; om, oromucosal; PB, phenobarbital; PCPC, Pediatric Cerebral Performance Category Scale; Quotient; SEF, status‐epilepticus‐associated‐with‐fever; sp, suppository; SPT, steroid pulse therapy; T1WI, T1 weighted image; T2WI, T2 weighted image; Thi, thiamylal; TTM+BCT, targeted temperature management plus barbiturate coma therapy; y, year.

**TABLE 2:** Clinical background of the patients with DEE.

Patient no.	Sex	Age of onset	Epileptic syndrome	Seizure types	Additional clinical findings	Brain imaging findings	Birth history	Developmental history	Family history	Age at genetic test	Anti‐epileptic medicine at genetic test	Other comments
Patient 16	M	2m	—	FBTCS	Mild global developmental delay, Facial abnormality: nasal ridge, full cheek, eyebrows that are thin on the inside, brachycephaly, long philtrum	MRI (2m): hypointensity of changes after subependymal hemorrhage (T2WI) and similar hypointensity in left occipital lobe subcortex	GA: 37w6d BW: 2222 g (−1.73 SD, 4.2%tile) HT: 42 cm (−2.68 SD, 0.4%tile) HC: 31.7 cm (−0.94 SD,17.5%tile), asymmetrical SGA, APS: 3/5/8	ESID: DQ55 at 7m	None	10m	VPA, LEV	
Patient 17	M	5m	IESS	ES	Macrocephaly, slight intellectual disability	MRI (5m): normal	GA: 39w2d BW: 3780 g	Head control (3m), rolling over (4m), KSPD 2001: DQ88 at 1y5m	Autism (niece), epilepsy&cerebral palsy (nephew)	10m	VitB6, VPA	EEG: hypsarrhythmia at seizure onset
Patient 18	F	1m	—	Tonic FIAS GMS	Autism, severe intellectual disability, insomnia	MRI (4m): normal	GA: 40w0d BW: 3535 g	KSPD 2001: DQ22 at 5y8m	None	15y	VPA, CZP	
Patient 19	F	8y	—	Myoclonic astatic	Mild global developmental delay	MRI (40y): cerebellar atrophy	GA: 41w0d BW: 3050 g	Head control (4m), start to walk (13m), slight developmental delay was pointed out at a previous hospital doctor at 8y, MMSE‐28 (40y), HDS‐R 26 (40y), FAB 16 (40y)	None	40y	LTG, TPM, VPA, CLB, PER	
Patient 20	F	3m	—	Tonic	Severe intellectual disability	N/A	GA: 38w2d BW: 2674 g	Head control (8m), roll over (10m), no significant word (16y), developmental milestone was at the level of 8 months (16y)	None	17y	VPA, LTG	
Patient 21	F	1d	—	GTCS tonic clonic GMS	Severe intellectual disability, scoliosis	CT (4d): normal	GA: 41w0d BW: 2548 g (IUGR), APS: 9/9	KSPD 2001: DQ19 at 7y7m	None	12y	PB, ZNS	EEG: suppression burst pattern at seizure onset
Patient 22	M	13m	—	Tonic, myoclonic, FIAS	Severe intellectual disability	MRI (15m): periventricular white matter hyperintensity (T2WI), MRI (2y): atrophy of frontal, temporal, and parietal lobes, MRI (8y8m): thinning of the entire cerebrum	GA: 40w1d BW: 3354 g	Psychomotor developmental delay was noted from 8 months. No acquisition of development of sitting. No word and pursuit, bedridden (17y)	None	17y	VPA, CLB	
Patient 23	M	1d	—	Tonic FIAS	Severe intellectual disability	CT (day2): normal, MRI (day4): normal	GA: 40w0d BW: 2992 g	No head control, opisthotonus, no smiling at 1y3m	None	1y3m	CBZ, PB	
Patient 24	F	2m	—	FIAS FBTCS ES	At onset, generalized muscle weakness and severe generalized developmental delay, cafe au lait spots, axillary freckle‐like pigmented spots	MRI (2y2m): normal	GA: 38w0d BW: 2684 g APS: 9/10	Head control (2y), pursuiting (1y10m), roll over (2y), sitting (2y8m), no significant word&no walking (3y)	None	3m	VPA, PB, KBr, VitB6	
Patient 25	F	6m	IESS	ES	Severe generalized developmental delay	MRI (1y8m): normal	GA: 38w6d BW: 3500 g	Developmental delay was pointed out at the 4‐month checkup, turning over and sitting (1y8m), stand up (3y4m), walking (3y6m), no word (3y6m), KSPD: DQ33 (1y7m)	None	2y	VPA	
Patient 26	F	3m	—	Tonic	Severe intellectual disability	MRI (4m): normal	GA: 41w6d BW: 3290 g APS: 9/10	Psychomotor developmental delay was pointed out at the 4‐month checkup. KSPD: DQ33 at 1y10m	None	1y0m	CBZ, LEV	
Patient 27	F	6m	—	GTCSS FIAS	Acquired microcephaly (45 cm at 3y, 47 cm 5y), stereotypic movements (hand kneading motion)	Unknown	Unknown	Sitting (1y2m), the reaction to the surroundings has slowed down and the function of the fingers has decreased (1y5m)	None	17y	LEV, CZP	
Patient 28	M	7y	—	FIAS	Mild developmental delay	MRI (3y): arachnoid cyst, Chiari malformation type I	GA: 37w4d BW: 3646 g	Head control (3m), sitting (5m), standing up (10m), walking (1y5m), significant word(11m), KPSD: DQ70 (6y)	None	8y	LEV	
Patient 29	M	3y	—	FIAS atonic	Mild developmental delay	CT (3y5m): mild dilation of left Sylvian fissure, arachnoid cyst	GA: 38w6d BW: 2718 g	KSPD 2001: DQ68 (4y)	Epilepsy (Father)	5y	TPM. VPA	
Patient 30	M	6m	—	GTCS	Mild verbal developmental delay, afebrile status epilepticus (3 times)	MRI (5m): normal	GA: 39w2d BW: 2886 g	Head control (4m), rolling over (6m), sitting (8m), standing up (10m), walking (1y8m), ESID: DQ63 (1y9m)	None	7m	VPA, LEV	
Patient 31	M	6y	—	Myoclonic astatic	Severe intellectual disability, autism	CT (6y): normal, MRI (7y): normal	GA: 40w0d BW: 2600 g cesarean section because of decreased heart rate	Hypotonia, inability to walk and no word were noted at the health checkup (1y6m). Can walk but no speech (7y), KSPD 2001: DQ17 (6y)	None	7y	VPA, PER	
Patient 32	F	6y	—	FIAS tonic	Moderate intellectual disability	MRI (1y6m): normal	GA: 38w0d BW: 2938 g	Head control (4m), walking (1y3m), no word (22y)	None	22y	VPA, RFM	
Patient 33	F	8m	—	Tonic	Moderate intellectual Disability	MRI (1y): normal	GA: 38w0d BW: 2675 g	Head control (4m), rolling over (5m), walking (1y), KSPD 2001: DQ45 (12y)	None	1y1m	ZNS	
Patient 34	F	9m	—	FMS	Global developmental delay	MRI (1y1m): normal	GA: 39w3d BW: 3242 g HT: 47.5 cm HC: 33.5 cm	Head control (4m), walking (1y7m), no word (1y7m)	None	1y1m	VPA, TPM, PER	
Patient 35	M	5m	IESS	ES	Moderate intellectual disability	MRI (6m): normal	GA: 40w6d BW: 3900 g HC: 35 cm	Rolling over (7m), no sitting (10m), ESID: DQ69 (6m), DQ77 (8.5m)	None	6m	VPA	
Patient 36	M	12m	—	ES GTCS tonic	Severe intellectual disability	N/A	GA: 40w2d BW: 2800 g	Head control (3m), sitting (7m), walking (1y6m), a few word (25y)	None	25y	VPA, LTG, RFM	
Patient 37	M	2m	—	Tonic	Slight global developmental delay	MRI (2m): normal	GA: 40w0d BW: 3634 g, HT:49.0 cm HC: 34.5 cm, Emergency cesarean section for stoppage of labor	No sitting (9m), ESID: DQ80 (9m)	None	2m	LCM	
Patient 38	M	13m	—	Absence	Mild global developmental delay, HPP, elevated urinary PEA	MRI (6m): normal	GA: 37w5d BW: 2936 g, planned cesarean section	Head control (5m), no sitting (9m), KPSI: DQ60 (1y)	Epilepsy (mother, two elder brothers, four aunts)	1y5m	VitB6	
Patient 39	F	6y	—	GTCS FIAS	Mild global developmental delay	MRI (7y, 10y): normal	GA: 38w0d BW: 2532 g APS: 9/10	developmental delay was pointed out at a medical checkup (3y) KSPD: DQ54 (10y)	None	14y	CLB, CBZ	
Patient 40	M	2d	EIDEE & IESS	FIAS tonic ES	Global developmental delay	MRI (2m): normal	GA: 41w3d BW: 3736 g APS: 8/10	no pursuiting (4m), head control (5m), no rolling over, sitting and no word (9m)	None	3m	VGB, LEV, VPA	
Patient 41	F	0d	—	FIAS	Severe intellectual disability	MRI (3y9m): frontal lobe atrophy	GA: 39w5d, BW: 2655 g, HC: 31.2 cm, APS: 9/10	sitting (6y), holdable (12y), no word (12y), KSPD: DQ13 (3y)	None	12y	LTG	
Patient 42	M	3y	—	FIAS myoclonic	Mild intellectual disability	MRI (15y): normal	GA: 41w0d, BW: 3100 g, APS: 10/10	KSPD: DQ44 (15y)	None	15y	VPA, ESM, RFM, CZP, LEV	

Abbreviations: APS, Apgar score; BW, body weight; CBZ, carbamazepine; CLB, clobazam, CT, computed tomography; CZP, clonazepam; DQ, Developmental Quotient; DWI, diffusion weighted imaging; EEG, electroencephalogram; EIDEE, early infantile developmental and epileptic encephalopathy; ES, epileptic spasm; ESID, Enjoji scale of infant analytical development; ESM, ethosuximide; F, female; FAB, frontal assessment battery; FBTCS, focal to bilateral tonic‐clonic; FIAS, focal impaired awareness seizures; FLAIR, fluid attenuated inversion recovery; FMS, focal nonmotor seizure; FS, febrile seizure; GA, gestational age; GMS, generalized motor seizure; GTCS, generalized tonic‐clonic seizure; HC, head circumference; HDS‐R, revised Hasegawa's dementia scale; HPP, Hypophosphatasia; HSES, hemorrhagic shock and encephalopathy syndrome; HT, height; IESS, infantile epileptic spasms syndrome; KPSI, Karnofsky performance scale index; KSPD, Kyoto scale of psychological development; LCM, lacosamide; LEV, levetiracetam; LTG, lamotrigine; M, male; m, month; MMSE, mini mental state examination; MRI, magnetic resonance imaging; N/A, not accessed; PB, phenobarbital; PEA, phosphoethanolamine; PER, perampanel; RFM, rufinamide; SGA, small‐for‐gestational age; T1WI, T1 weighted image; T2WI, T2 weighted image; TPM, topiramate; VGB, vigabatrin; Vit, vitamin; VPA, valproic acid; y, year; ZNS, zonisamide.

### 3.2 Results of Genetic Tests for the Patients With SEF and DEE

Genetic tests performed for each individual patient are shown in Table S3. Genetic variants explaining the clinical presentation were detected in four of the 15 patients with SEF and in 17 of the 27 patients with DEE. In patients with SEF, the variants were all found in *SCN1A* (*n* = 4). In patients with DEE, the variants were found in *STXBP1* (*n* = 2), *ALPL* (*n* = 1), *CDKL5* (*n* = 1), *CSNK2B/TUBB* (*n* = 1), *DEPDC5* (*n* = 1), *GABRB3* (*n* = 1), *GNAO1* (*n* = 1), *KCNB1* (*n* = 1), *KCNQ2* (*n* = 1), *PACS1* (*n* = 1), *PIK3CA* (*n* = 1), *PTEN* (*n* = 1), *SCN1A* (*n* = 1), *SCN8A* (*n* = 1), *SHANK3* (*n* = 1), and *TRRAP* (*n* = 1; Table 3).

**TABLE 3:** Summary of genetic tests performed on each patient.

Patient No.	Identified method	gene			Variant	rs number	Inheritance	Zygosity	gnomAD frequency	ACMG guideline	HGMD pro 2022.4 (number of references)
				**SEF/AE**							
Patient 4	Panel sequencing	*SCN1A*			NM_001165963.3:c.2194C>T,p.(Q732*)		De novo	Hetero	0	Pathogenic (PVS1+PS2+PM2)	DM(1)
Patient 5	Commercial	*SCN1A*			NM_001165963.3:c.3637C>T,p.(R1213*)	rs794726710	De novo	Hetero	0	Pathogenic (PVS1+PS2+PM2)	DM(13)
Patient 7	Commercial	*SCN1A*			NM_00165963.3:c.4398C>A,p.(F1466L)		Unknown	Hetero	0	VUS (PM2+PM5 +PP3)	Novel
Patient 9	WES	*SCN1A*			NM_001165963.3:c.1‐74_1‐54del		De novo	Hetero	0	Likely pathogenic (PS2+PM2)	Novel
				**DEE**							
Patient 16	Microarray	*CSNK2B, TUBB*			arr[GRCh37]6p21.33p21.32(30578599×2,30630793_32430238×1,32460307×2)		Unknown	Hetero	N/A	N/A	N/A
Patient 17	Panel sequencing	*PTEN*			NM_00314.4:c.737C>T,p.(P246R)	rs587782350	De novo	Hetero	0	Likely pathogenic (PS2+PM2+PP3)	DM(16)
Patient 18	Panel sequencing	*CDKL5*			NM_003159.3:c.2635_2636del,p.(L879Efs*30)	rs61753251	De novo	Hetero	0	Pathogenic (PVS1+PS2+PM2)	DM(12)
Patient 20	Panel sequencing	*STXBP1*			NM_003165.6:c.734A>G,p.(H245R)	rs587784453	De novo	Hetero	0	Likely pathogenic (PS2+PM2+PP3)	DM(3)
Patient 23	Panel sequencing	*KCNQ2*			NM_172107.4:c.868G>A,p.(G290S)	rs1057516098	De novo	Hetero	0	Likely pathogenic (PS2+PM2+PP3)	DM(5)
Patient 24	Panel sequencing	*DEPDC5*			NM_001242896.3:c.58+2_58+3insCA		De novo	Hetero	0	Pathogenic (PVS1^a^+PS2+PM2)	Novel
Patient 25	Panel sequencing	*KCNB1*			NM_004975.4:c.1219C>T,p.(L407F)		De novo	Hetero	0	Likely pathogenic (PS2+PM2+PP3)	Novel
Patient 26	WES	*GABRB3*			NM_000814.6:c.778C>A,p.(L260M)		De novo	Hetero	0	Likely pathogenic (PS2+PM2+PP3)	Novel
Patient 27	WES	*TRRAP*			NM_003496.4:c.10537G>A,p.(E3513K)		De novo	Hetero	0	Likely pathogenic (PS2+PM2+PP3)	Novel
Patient 28	Panel sequencing	*PIK3CA*			NM_006218.4:c.2740 = /G>A,p.(Gly914 = /Arg)	rs587776932	De novo	Hetero	0	Likely pathogenic (PS2+PM2+PP3)	DM(13)
Patient 30	Commercial	*SCN1A*			NM_001165963.3:c.4072T>C,p.(Trp1358Arg)		Unknown	Hetero	0	VUS (PM2+PP3)	DM?(4)
Patient 31	WES	*SHANK3*			NM_001372044.2:c.4461dup,p.(P1488Afs*62)		De novo	Hetero	0	Pathogenic (PVS1+PS2+PM2)	Novel
Patient 35	Panel sequencing	*SCN8A*			NM_014191.4:c.2882T>G,p.(M961R)		De novo	Hetero	0	Likely pathogenic (PS2+PM2+PP3)	Novel
Patient 37	Panel sequencing	*PACS1*			NM_018026.4:c.607C>T,p.(R203W)	rs398123009	De novo	Hetero	0.000006572	Pathogenic (PS2+PS3+PP3)	DM(42)
Patient 38	Panel sequencing	*ALPL* ^b^			NM_000478.6:c.1559delT,p.(L520Rfs*86)	rs387906525	From mother	Hetero	0.00001314	Likely pathogenic (PVS1+PM2)	DM(20)
Patient 40	Panel sequencing	*STXBP1*			NM_003165.6:c.325+1G>A		De novo	Hetero	0	Pathogenic (PVS1+PS2+PM2)	Novel
Patient 42	Panel sequencing	*GNAO1*			NM_020988.3:c.680C>T,p.(A227V)	rs797045599	De novo	Hetero	0	Likely pathogenic (PS2+PM2+PP3)	DM(5)

a: This variant causes exon 2 skipping, confirmed by analysis of blood‐derived cDNA. Figure result is shown in Figure S1.

b: It is controversial whether this heterozygous variant is the cause of the epilepsy in this case. Similar to our case, heterozygotes for truncating mutations in ALPL have been also previously reported (Michigami et al. 2005; Kitoh et al. 2022). However, we concluded that there was an association between this variant and epilepsy in this case, based on the low blood ALP, high urinary PEA levels, and the significant effect of vitB6 on the epileptic seizures.

Abbreviations: ACMG, American College of Medical Genetics and Genomics; DM, disease‐causing mutations; gnomAD, Genome Aggregation Database; HGMD, Human Gene Mutation Database; N/A, not accessed; WES, whole exome sequencing.

Thirteen of the 15 patients with SEF and 24 of the 27 patients with DEE underwent original panel sequencing. There was no significant difference in the number of original panel sequencings performed in both groups (*p* = 1.00; Fisher's exact test; Table 4). Three of 15 patients with SEF and 12 of 27 patients with DEE underwent whole‐exome sequencing. There was no significant difference in the number of WES analyses performed in both groups (*p* = 0.18; Fisher's exact test; Table 4). Two of the 15 patients with SEF and six of the 27 patients with DEE underwent microarray analysis. There was no significant difference in the number of microarrays performed in both groups (*p* = 0.69; Fisher's exact test; Table 4). Two of the 15 patients with SEF and two of the 27 patients with DEE underwent other genetic testing, including direct sequencing for *PIK3CA* and commercial‐based panel sequencing for Dravet syndrome. There was no significant difference in the number of other genetic tests performed in both groups (*p* = 0.61, Fisher's exact test; Table 4).

**TABLE 4:** Comparison of the number of each genetic test performed on both groups.

	SEF (*n* = 15)	DEE (*n* = 27)	*p*‐value
Original panel sequencing	13	24	1.00
Positive (%)	1 (7.7)	12 (50.0)	—
Whole exome sequence	3	12	0.18
Positive (%)	1 (33.3)	3 (25.0)	—
			
Microarray	2	6	0.69
Positive (%)	0 (0)	1 (16.7)	—
Others			
Direct sequence	2	2	0.61
Commercial‐based genetic testing			
Positive (%)	2 (100)	1 (50.0)	—

Abbreviations: DEE, developmental and epileptic encephalopathy; SEF, status epilepticus associated with fever.

For Patients 7 and 30, the parents’ DNAs were not obtained; therefore, we could not confirm that the variants were de novo in their occurrence. The variant (NM_001165963.3:c.4398C>A,p.F1466L) detected in Patient 7 was not registered in the Genome Aggregation Database (gnomAD). Multiple in silico analyses (e.g., Mutation Taster, Polyphen‐2, SIFT, and CADD) predicted that the variant was damaging. A variant, in which the same amino acid residue is changed to serine (p.F1466S), is reportedly pathogenic (Lindy et al. 2018). Patient 7 was clinically diagnosed with Dravet syndrome, and no variants were identified in other genes associated with the syndrome, such as *SCN1B*, *SCN2A*, and *GABRG2*. Therefore, although the variant is classified as a variant of uncertain significance according to the ACMG guidelines, it was considered to be the likely cause of the disease.

The variant NM_001165963.3.4072T>C (p.W1358R) detected in patient 30 was previously reported as a de novo occurrence in a patient with Dravet syndrome (Zuberi et al. 2011). Additionally, this variant is not registered in the gnomAD database and was predicted to be damaging by multiple in silico analyses. Patient 30 was also clinically diagnosed with Dravet syndrome. As this variant has been previously reported as pathogenic, it was determined to be the cause of the disease in this case.

### 3.3 Comparison of the Results of Genetic Tests for the Patients With SEF and DEE

Four of 15 patients with SEF/AE and 17 of 27 patients with DEE had a genetic variant that explained their clinical presentation. The detection rate of genetic variants was higher in patients with DEE (63.0%) than in those with SEF (26.7%), although there was no statistically significant difference (*p* = 0.05, Fisher's exact test).

In both the SEF and DEE groups, no statistically significant differences were found between patients who tested positive for genetic mutations (genetic test+) and those who tested negative (genetic test−) across various clinical factors. Imaging abnormalities, such as those detected by computed tomography or magnetic resonance imaging, were observed in 50.0% of cases in both SEF groups (*p* = 1.0) and 13.3% versus 44.4% in the DEE group (*p* = 0.15). Past medical history showed a difference of 100% versus 45.5% in SEF (*p* = 0.10) and 100% versus 100% in DEE (*p* = 1.0). Developmental delays were present in 50.0% versus 36.4% in SEF (*p* = 1.0) and 100% versus 100% in DEE (*P* = 1.0). Similarly, family history showed no significant difference, with 25.0% versus 45.5% in SEF (*p* = 0.60) and 11.8% versus 10.0% in DEE (*p* = 1.0). These analyses were conducted using Fisher's exact test.

## 4 Discussion

We investigated the disease‐associated gene variants in patients with SEF and DEE. In addition, we compared the variants among patients with SEF and DEE to reveal the differences between SEF and DEE in terms of the influence of each genetic variant on the cause of the disease. The incidence of SEF is particularly low outside of East Asia, making it difficult to accumulate cases, and attention is often focused on environmental factors, such as the cause of infectious diseases. To the best of our knowledge, this is the first study to elucidate the different single genetic variants of SEF and compare them to those of DEE. The present study suggests that SEF may differ from DEE in terms of the diversity of single‐gene variants and the amount of variants that can be attributed to a specific gene.

Numerous genetic etiologies of DEE have been reported to date (Specchio and Curatolo 2021). Single or multiple genetic mutations combined with environmental factors are found in approximately 30%–70% of epilepsy cases (Wang et al. 2021; Çapan et al. 2024; Vetri et al. 2024). Our study identified causative genetic variants in 63% of DEE cases, aligning with previous studies. In the control group, several novel variants were found to be associated with DEE (patients 7, 9, 16, 24, 25, 26, 27, 31, 35, and 40), most of which were confirmed as de novo occurrences and classified as “pathogenic” or “likely pathogenic” according to the ACMG/AMP guidelines (Richards et al. 2015). Some of these variants were clinically and genetically important (Patients 16 and 17).

The SEF is closely related to the AE. Although some studies have reported a single‐gene mutation variant of SE with epilepsy (Wang et al. 2021; Neubauer and Hahn 2014; Bhatnagar and Shorvon 2015), mutation variants of SEF are scarce because many studies have focused on the source of fever, such as pathogenic microorganisms (Han and Han 2023). Contrastingly, AE is often an infection‐triggered emergency syndrome. It is assumed that the immune response, metabolism, and neuronal excitation are important triggers of AE (Mizuguchi et al. 2023). Furthermore, environmental factors, such as pathogenic microorganisms (e.g., influenza virus, human herpesvirus‐6), are important first triggers; however, genetic factors have also been reported to be the underlying etiology in some patients with AE. Regarding infection‐triggered AE, there are previously published case reports, case series, or studies on types of single‐gene variants within a particular disease (Saitoh et al. 2012, 2015; Shibata et al. 2020; Van et al. 2021; Kobayashi et al. 2010, 2012, 2013; Fukasawa et al. 2015; Neilson et al. 2009; Xavier et al. 2020; Denier et al. 2014; Ohashi et al. 2021; Mancardi et al. 2021; Shimada et al. 2018; Oláhová et al. 2018; Torisu et al. 2006; Isobe et al. 2022; Saito et al. 2022; Kurahashi et al. 2018; Tamhankar et al. 2020; Okuzono et al. 2019; Pezzani et al. 2018; Belal et al. 2018; Schmelzer et al. 2018; Merwick et al. 2013; Zhang et al. 2022; Schon et al. 2003; Ruitenbeek et al. 1995; Hidaka et al. 2006; Kara et al. 2022; Hanafusa et al. 2023). The genes encoding ion channels, that is, *SCN1A* and *SCN2A*, are the most commonly reported (Saitoh et al. 2012, 2015; Shibata et al. 2020; Van et al. 2021; Kobayashi et al. 2010; Fukasawa et al. 2015; Kobayashi et al. 2012; Hanafusa et al. 2023). In the present study, although many rare variants were found in DEE, only variants of *SCN1A* were found in SEF. Our study demonstrated that sodium channels are a critical factor in SEF. Interestingly, none of the patients with DEE in our study had a history of SEF, and the only gene common to both SEF and DEE cases was *SCN1A*. Therefore, we hypothesized that SEF and DEE would have different onset mechanisms. In DEE, there is a relatively strong genetic predisposition, while in SEF, environmental factors, such as the pathogenicity of infectious diseases, may play a role alongside the genetic predisposition associated with *SCN1A* mutations (Mizuguchi et al. 2007, 2021). Recent studies have reported associations between genetic polymorphisms and FSs or AE (Shibata et al. 2022; Skotte et al. 2022; Dimitrijevic et al. 2022). It is possible that the role of genetic polymorphisms in SEF will become clearer with further research. However, determining whether environmental factors or genetic predisposition exert a more significant influence requires additional investigation.

This study had some limitations. First, it had a retrospective, single‐center design, limiting the generalizability of its results. Second, as the convulsion time was estimated based on interviews with family members, the accuracy of reporting cannot be confirmed. Third, our study included a small number of patients because of its single‐center design. Despite these limitations, our study provides new insights into the pathophysiology of SEF and DEE.

In conclusion, analysis of patients with DEE revealed a wide variety of causative genes for DEE, whereas in SEF cases, only *SCN1A* variants were detected. Our study is the first to suggest that there is a difference in the detection rate of single gene variants of SEF, compared to that of the single gene variants of DEE, thereby implying that SEF may have a different pathophysiology in terms of single gene variants. Our findings can be further validated by accumulating additional databases of SEF cases to demonstrate the respective proportions of single gene variants amongst these diseases. Panel sequencing is highly useful for identifying genetic variants, but in some cases, pathogenic variants have been detected through WES rather than panel sequencing. As the cost of WES continues to decrease, it may become a more accessible and effective tool for genetic testing, offering broader diagnostic potential.

## Author Contributions

**Hiroaki Hanafusa**: conceptualization, writing–original draft, methodology, data curation, investigation, formal analysis. **Hiroshi Yamaguchi**: conceptualization, investigation, funding acquisition, writing–original draft, methodology, validation, visualization, writing–review and editing, formal analysis, project administration, data curation, supervision. **Naoya Morisada**: data curation, writing–review and editing, methodology, resources, formal analysis. **Ming Juan Ye**: methodology, writing–review and editing, data curation, resources, formal analysis. **Shizuka Oikawa**: writing–review and editing, supervision, data curation. **Shoichi Tokumoto**: Writing–review and editing, supervision, data curation. **Masahiro Nishiyama**: writing–review and editing, supervision, data curation. **Kandai Nozu**: Writing–review and editing, supervision, data curation. **Hiroaki Nagase**: conceptualization, investigation, writing–review and editing, supervision, methodology, project administration, resources.

## Ethics Statement

This retrospective clinical observational study was approved by the Ethics Committee of Kobe University (2022‐86). Informed consent was obtained from the parents of all patients. All the experiments in this study were conducted in accordance with the relevant guidelines and regulations, or in accordance with the Declaration of Helsinki. Written informed consent for publication of identifying images or other personal or clinical details was obtained from the parents or legal guardians of any participant under the age of 18.

## Conflicts of Interest

The authors declare no conflicts of interest.

### Peer Review

The peer review history for this article is available at https://publons.com/publon/10.1002/brb3.70279

## Supporting information



TABLE S1 Primer list for *PIK3CA*.

TABLE S2 The 331 genes included in the original gene panel.

TABLE S3 Genetic tests performed for each individual patient.

**FIGURE S1** cDNA analysis of *DEPDC5* in patient 24. We performed a cDNA analysis and found a short band in addition to the usual band in the patient, and exon 2 skipping was confirmed by sequencing analysis.

## Data Availability

The datasets generated and/or analyzed during the current study are available in the Harvard Dataverse repository, “[https://doi.org/10.7910/DVN/NE6ASB].” Further data used in this study will be made available to other researchers upon reasonable request.
