# Supplementary material for: Identification of Genetic Variants in Status Epilepticus Associated With Fever
Source: Brain Behav. 2025 Feb 6;15(2):e70279. doi: 10.1002/brb3.70279 (PMC11802276; doi:10.1002/brb3.70279)
Supplement: Supplementary file 1 — TABLE S1 Primer list for PIK3CA. [file BRB3-15-e70279-s002.docx]

**Table S1. Primer list for *PIK3CA***

| Exon2a | Forward | AGCCTAATCAAGTCAAACTATGGA |
| --- | --- | --- |
|  | Reverse | TCACGGTTGCCTACTGGTTC |
| Exon2b | Forward | CGTGAGGCTACATTAATAACCATAA |
|  | Reverse | GGTGTTAAAAATAGTTCCATAGTTCGA |
| Exon3 | Forward | ACAGAGTTCCCTGTTTGCAA |
|  | Reverse | CCCTGCCTTCAAGAAGCTTAC |
| Exon4 | Forward | TTGGGAATGATCTGGCAGCC |
|  | Reverse | GGCGAGAGTGAGATTCCGTC |
| Exon5 | Forward | TGATTGATCTTGTGCTTCAACGT |
|  | Reverse | TGGATGTTCTCCTAACCATCTGA |
| Exon6 | Forward | TCTGAACAAAAATTCCGTGGTT |
|  | Reverse | AATGGGGTCTTGCTTTGTTGC |
| Exon7 | Forward | AGTGCCTTTTCCAATCAATCTCT |
|  | Reverse | TCCTGAAGCTCTCCCAAGGT |
| Exon8-9 | Forward | TCCTTTTGGGGAAGAAAAGTGT |
|  | Reverse | TGCAATATTGGTCCTAGAGTTCA |
| Exon10 | Forward | AGATTGGTTCTTTCCTGTCTCTGA |
|  | Reverse | TAGGTATGGTAAAAACATGCTGAGA |
| Exon11 | Forward | TTTCCTTTTGTGTTCTTTGCCA |
|  | Reverse | TTATGTGGACTTTCTGAGAGAAAAC |
| Exon12 | Forward | TCAGAAGTTAAGGCAGTGTTTTAGA |
|  | Reverse | GCATAAAACTAGTTAGTGCAGTAGGT |
| Exon13 | Forward | ACCTACTGCACTAACTAGTTTTATGC |
|  | Reverse | GCAAAAGTTGAGAAGCTCATCACT |
| Exon14 | Forward | ACTACCTGAAACTCATGGTGGT |
|  | Reverse | GAGCCTGCATGACGTGTCTA |
| Exon15 | Forward | TGCTCTGTGTTGTAGAAACCCT |
|  | Reverse | TGAGGGTAGGAGAATGAGAGAGA |
| Exon16 | Forward | TGAGGTGAAAGTTGTAAATCTTTGT |
|  | Reverse | ACATGCATATTTCAAAGGTCAAGACA |
| Exon17 | Forward | AAGAAATCAGAATATTGCTTTCCTGA |
|  | Reverse | ATTTAGCTATTCTAAGTAAGAGGAGGA |
| Exon18 | Forward | TCCAGGAGTATGTTTATCACACCA |
|  | Reverse | ACACAAACACCGACAGACTCA |
| Exon19-20 | Forward | TGGAAACTTGCACCCTGTTT |
|  | Reverse | ACCACACCCAGCCAACATTT |
| Exon21 | Forward | GCTTTGTCTACGAAAGCCTCTC |
|  | Reverse | TGTTCTTGCTGTAAATTCTAATGCTGT |
|  |  |  |
