# Supplementary material for: Identification of Genetic Variants in Status Epilepticus Associated With Fever
Source: Brain Behav. 2025 Feb 6;15(2):e70279. doi: 10.1002/brb3.70279 (PMC11802276; doi:10.1002/brb3.70279)
Supplement: Supplementary file 2 — TABLE S2 The 331 genes included in the original gene panel. [file BRB3-15-e70279-s001.docx]

**Table S2. The 331 genes included in the original gene panel**

*AARS, ABAT, ACADL, ACADM, ACADS, ACY1, ADAM22, ADAM23, ADAR, ADCK3, ADGRV1, ADORA2A, ADSL, AKT3, ALAD, ALAS2, ALDH4A1, ALDH7A1, ALG13, ALPL, AMT, ANK3, APBA1, APBA2, ARHGEF15, ARHGEF9, ARSA, ARX, ASAH1, ASNS, ASPM, ATP13A2, ATP1A3, ATP6AP2, ATP7A, ATP7B, BCKDK, BRAT1, BTD, CACNA1A, CACNA1E, CACNA2D1, CACNA2D2, CACNB4, CASK, CASR, CBL, CBS, CDKL5, CHD2, CHD5, CHL1, CHRNA2, CHRNA4, CHRNA7, CHRNB2, CLCN2, CLCN4, CLN3, CLN5, CLN6, CLN8, CLVS2, CNTN5, CNTNAP2, COL4A1, COX10, CPA6, CPLX1, CPOX, CPT1A, CPT1B, CPT2, CRIPT, CRMP1, CSNK1G1, CSNK1G2, CSTB, CTSA, CTSD, CTSF, CTSO, D2HGDH, DCX, DEPDC5, DLGAP1, DLGAP2, DLGAP3, DLX1, DLX2, DNAJC5, DNAJC6, DNM1, DOCK7, DYRK1A, EEF1A2, EFCAB2, EFHC1, EPHA6, EPHB2, EPM2A, EPN1, ERBB4, FARS2, FASN, FECH, FH, FLNA, FOLR1, FOXG1, FRRSIL, FZD9, GABBR2, GABRA1, GABRA2, GABRA5, GABRB1, GABRB2, GABRB3, GABRD, GABRG2, GABRG3, GAD1, GAMT, GATM, GBA, GCSH, GFAP, GLDC, GLYCTK, GNAI1, GNAO1, GOSR2, GPHN, GRIN1, GRIN2A, GRIN2B, GRIN2D, GRN, GTPBP3, HADH, HADHA, HADHB, HCN1, HCN2, HCN4, HDAC4, HEXA, HEXB, HFE, HLCS, HMBS, HNRNPU, IDH1, IDS, IL1B, IL1RN, IMPA2, IQSEC2, ITPA, KANSL1, KCNA1, KCNA2, KCNB1, KCNC1, KCND2, KCNH5, KCNJ10, KCNJ11, KCNMA1, KCNQ2, KCNQ3, KCNT1, KCNT2, KCNV2, KCTD7, KIAA1456, KLHL17, KPNA7, L2HGDH, LGI1, LIAS, LPHN2, MAGEL2, MAGI2, MAPK10, MAPK8, MBD5, MCCC2, MECP2, MEF2C, MFSD8, MLC1, MMADHC, MOCS1, MOCS2, MOCS3, MTHFR, MTMR1, MTOR, MTR, MTRR, NCAM2, NECAP1, NEDD4L, NEU1, NHLRC1, NRXN1, NTNG1, OCLN, OPHN1, OR10H2, OTC, OTX1, PAFAH1B1, PAH, PC, PCDH19, PDHX, PDK1, PDSS2, PDYN, PHGDH, PIGA, PIGO, PIGQ, PLCB1, PNKP, PNPO, POLG, PPOX, PPP2R2C, PPT1, PRICKLE1, PRICKLE2, PRKCZ, PRODH, PRRT2, PSAT1, PTEN, PURA, QARS, RANBP2, RANGAP1, RCOR1, REL, RHOBTB2, RNASEH2A, RNASEH2B, RNASEH2C, RRM2B, RYR3, SAMHD1, SCARB2, SCN1A, SCN1B, SCN2A, SCN2B, SCN3A, SCN8A, SCN9A, SEMA3A, SEMA3E, SETBP1, SH3GL2, SHROOM2, SIK1, SLC12A5, SLC13A5, SLC19A3, SLC1A1, SLC1A3, SLC22A5, SLC25A13, SLC25A15, SLC25A20, SLC25A22, SLC25A29, SLC2A1, SLC35A2, SLC46A1, SLC6A1, SLC6A8, SLC9A6, SMARCA2, SNPH, SP1, SPTAN1, SRGAP2, SRP9, SRPX2, ST3GAL3, ST3GAL5, STK39, STRADA, STX1A, STX1B, STXBP1, STYXL1, SUOX, SV2A, SV2B, SYN1, SYN2, SYNGAP1, SYNJ1, SYT2, TBC1D24, TBL1XR1, TCF4, TLR3, TNK2, TPP1, TREX1, TSC1, TSC2, TSEN54, TTN, TWNK, UBE2A, UBE3A, UPB1, UROD, UROS, WDR62, WWOX, ZEB2, ZMYND8, ZNF182, ZNF532, ZNF536*
