# Supplementary material for: Identification of Genetic Variants in Status Epilepticus Associated With Fever
Source: Brain Behav. 2025 Feb 6;15(2):e70279. doi: 10.1002/brb3.70279 (PMC11802276; doi:10.1002/brb3.70279)
Supplement: Supplementary file 3 — TABLE S3 Genetic tests performed for each individual patient. [file BRB3-15-e70279-s004.docx]

| **Table S3.** **Genetic tests performed for each individual patient.** | | | | |
| --- | --- | --- | --- | --- |
| Patient No. | Original panel sequencing | Direct sequence or Commercial-based genetic testing | Microarray | Whole exome sequence |
| SEF/AE |  |  |  |  |
| Patient 1 | ✓ |  |  |  |
| Patient 2 | ✓ |  |  |  |
| Patient 3 | ✓ |  | ✓ | ✓ |
| Patient 4 | ✓ |  |  |  |
| Patient 5 |  | ✓ |  |  |
| Patient 6 | ✓ |  |  |  |
| Patient 7 |  | ✓ |  |  |
| Patient 8 | ✓ |  | ✓ |  |
| Patient 9 | ✓ |  |  | ✓ |
| Patient 10 | ✓ |  |  |  |
| Patient 11 | ✓ |  |  | ✓ |
| Patient 12 | ✓ |  |  |  |
| Patient 13 | ✓ |  |  |  |
| Patient 14 | ✓ |  |  |  |
| Patient 15 | ✓ |  |  |  |
|  |  |  |  |  |
| DEE |  |  |  |  |
| patient 16 | ✓ |  | ✓ |  |
| patient 17 | ✓ |  |  |  |
| patient 18 | ✓ |  |  |  |
| patient 19 | ✓ |  |  | ✓ |
| patient 20 | ✓ |  |  |  |
| patient 21 | ✓ |  | ✓ | ✓ |
| patient 22 | ✓ |  | ✓ | ✓ |
| patient 23 | ✓ |  |  |  |
| patient 24 | ✓ |  |  |  |
| patient 25 | ✓ |  |  |  |
| patient 26 |  |  |  | ✓ |
| patient 27 |  |  |  | ✓ |
| patient 28 | ✓ | ✓ |  |  |
| patient 29 | ✓ |  |  | ✓ |
| patient 30 |  | ✓ |  |  |
| patient 31 | ✓ |  |  | ✓ |
| patient 32 | ✓ |  |  | ✓ |
| patient 33 | ✓ |  | ✓ | ✓ |
| patient 34 | ✓ |  |  |  |
| patient 35 | ✓ |  |  |  |
| patient 36 | ✓ |  | ✓ | ✓ |
| patient 37 | ✓ |  | ✓ | ✓ |
| patient 38 | ✓ |  |  |  |
| patient 39 | ✓ |  |  |  |
| patient 40 | ✓ |  |  |  |
| patient 41 | ✓ |  |  |  |
| patient 42 | ✓ |  |  | ✓ |
