# Supplementary figures and images for: Identification of Genetic Variants in Status Epilepticus Associated With Fever
Source: Brain Behav. 2025 Feb 6;15(2):e70279. doi: 10.1002/brb3.70279 (PMC11802276; doi:10.1002/brb3.70279)

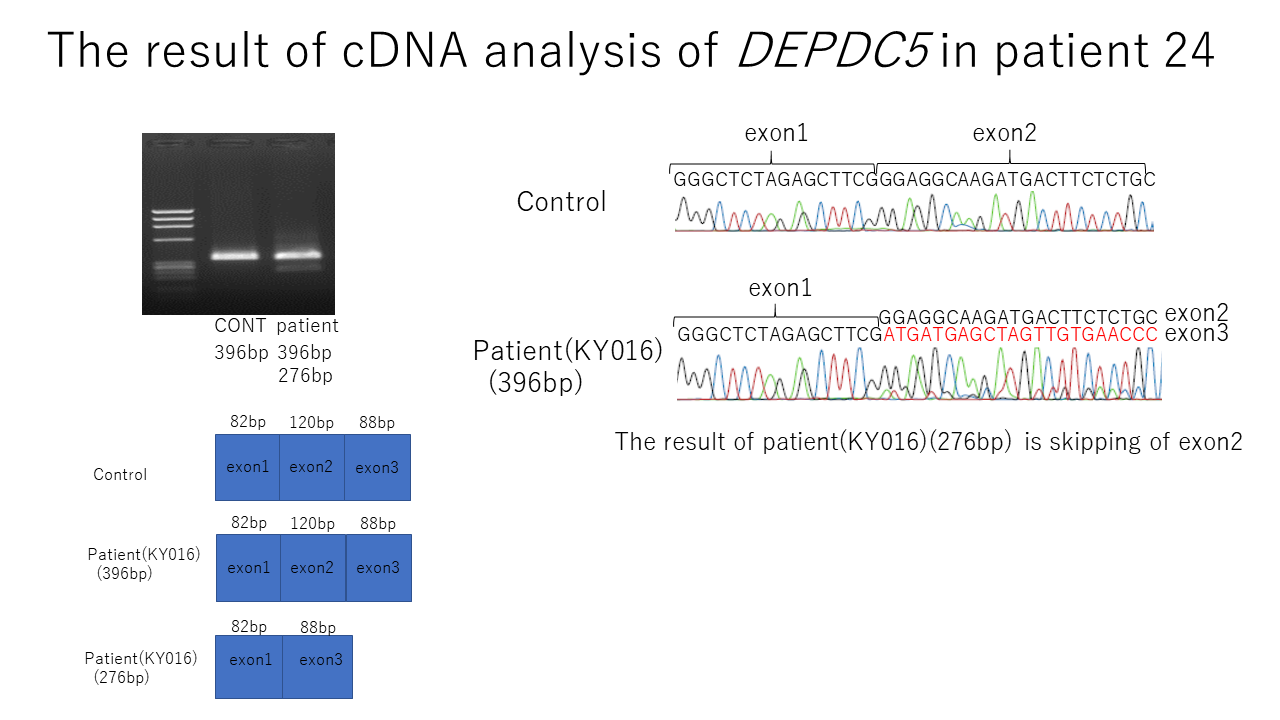

Supplement: Supplementary file 4 — FIGURE S1 cDNA analysis of DEPDC5 in patient 24. We performed a cDNA analysis and found a short band in addition to the usual band in the patient, and exon 2 skipping was confirmed by sequencing analysis. [file BRB3-15-e70279-s003.tif]
